# PICS bags safely store unshelled and shelled groundnuts in Niger

**DOI:** 10.1016/j.jspr.2017.03.007

**Published:** 2017-05

**Authors:** D. Baributsa, I.B. Baoua, O.N. Bakoye, L. Amadou, L.L. Murdock

**Affiliations:** a Department of Entomology, Purdue University, West Lafayette, IN, 47907, USA; b Université de Maradi, BP 465, Maradi, Niger; c Institut National de la Recherche Agronomique du Niger (INRAN), BP 240, Maradi, Niger

**Keywords:** Groundnuts, Hermetic storage, PICS bags, Insect pests, Niger

## Abstract

We conducted an experiment in Niger to evaluate the performance of hermetic triple layer (Purdue Improved Crop Storage- PICS) bags for the preservation of shelled and unshelled groundnut *Arachis hypogaea* L. Naturally-infested groundnut was stored in PICS bags and woven bags for 6.7 months. After storage, the average oxygen level in the PICS bags fell from 21% to 18% (v/v) and 21%–15% (v/v) for unshelled and shelled groundnut, respectively. Identified pests present in the stored groundnuts were *Tribolium castaneum* (Herbst), *Corcyra cephalonica* (Stainton) and *Cryptolestes ferrugineus* (Stephens). After 6.7 months of storage, in the woven bag, there was a large increase in the pest population accompanied by a weight loss of 8.2% for unshelled groundnuts and 28.7% for shelled groundnut. In PICS bags for both shelled and unshelled groundnuts, by contrast, the density of insect pests did not increase, there was no weight loss, and the germination rate was the same compared to that recorded at the beginning of the experiment. Storing shelled groundnuts in PICS bags is the most cost-effective way as it increases the quantity of grain stored.

## Highlights

• Weight loss of grain stored in woven bags is higher in shelled groundnuts compared to unshelled groundnuts.
• No storage loss is observed when shelled and unshelled groundnuts are stored in hermetic bags.
• Storing shelled groundnuts in hermetic bags is effective and more economical.

## 1 Introduction

Groundnut (*Arachis hypogaea* L.) is one of the most important cash crops in West Africa. Groundnuts generates about 60 percent of farmers' income in the region, and as much as 80% in Senegal (Ntare et al., 2004, Sylla, 2010). West Africa produces about 5.87 million tons of groundnuts. This accounts for about 67% of Africa's total production and 16% of world production (Ntare et al., 2004). Groundnuts are grown in West Africa as a source of protein (between 24 and 35% depending on the variety) and carbohydrates (Rehm and Espig, 1991). They are also an important crop because of their high content in oil, which is in the range of 38–50% (Ribier and Rouzière, 1995). Frequently grown by smallholder farmers, groundnuts are often considered a woman's crop. In Niger, groundnut is the fourth most important crop. It is cultivated on more than 700,000 ha with an estimated 402,000 tons produced each year (M.A, 2015). In Niger, groundnut is used to produce cooking oil, and its paste is used to prepare local dishes.

Despite its economic and food security importance, groundnut has postharvest storage challenges due mainly to pests and aflatoxin. Most smallholder farmers and especially those in Africa store groundnuts in unshelled form (Nautiyal, 2002, Ntare et al., 2004). Although unshelled groundnuts are bulky, keeping them in the shell as long as possible is an effective approach to reduce insect infestation during storage (Ranga Rao et al., 2010). Though it is more economical to store shelled groundnuts, the grains are more susceptible to insect attacks. Stored groundnuts are infested by numerous insect pests including: *Caryedon serratus* (Olivier), *Tribolium confussum* (Jacquelin du Val), *Corcyra cephalonica* (Stainton), *Tribolium castaneum* (Herbst), *Cryptolestes ferrugineus* (Stephens), *Oryzaephilus surinamensis* (L.) (Delobel and Matokot, 1990, Ndiaye, 1991, Ranga Rao et al., 2010, Guèye et al., 2011, Raoul and Léonard, 2013, Baoua et al., 2015). The weevil *C. serratus* is the most serious storage pest in some regions (Delobel, 1989, Gagnepain and Rasplus, 1989). *C. serratus* and *C. cephalonica* can cause storage losses as high as 70 and 80% in India (Harish et al., 2012); 9.3–62.8% in Congo (Matokot et al., 1987); and about 16% in Niger (Baoua et al., 2015).

In West Africa, groundnuts are stored in many types of containers including clay pots, jute and woven plastic bags, mud silos, plastic and metal drums, and glass jars (Masters et al., 2013). These containers provide little or no protection against insect pests. Different approaches are used to manage pests during groundnuts storage. Drying unshelled groundnut below 7% helps reduce insect infestations and incidence of aflatoxin. Natural or synthetic insecticides may help preserve groundnut quality during storage (Guèye et al., 2011, Conway, 1975, Putnam et al., 1991). Insecticide treatment may be required for groundnuts destined for export (APEDA, 2015). Several chemicals are recommended for insect control including fumigants and, powdered and liquid insecticides (Ranga Rao et al., 2010).

Hermetic storage such as the Purdue Improved Crop Storage (PICS™) bags have shown to be effective in preserving many cereal and legume crops in Sub-Saharan Africa (Baributsa et al., 2010, Baoua et al., 2014a, Murdock et al., 2012). PICS bags serve both as storage containers and also as postharvest storage pest management tools. These bags have already been tested for the protection of groundnuts against *C. serratus* and aflatoxin contamination in India (Sudini et al., 2015). After 4 months of storage in small custom-made hermetic bags holding 2 kg, groundnut quality including germination was well maintained. No study has been conducted in West Africa on the effectiveness of hermetic PICS bags under Sahelian conditions. The purpose of the present study were: (i) to evaluate the performance of PICS bags for storing naturally-infested shelled and unshelled groundnuts in Niger, and; (ii) identify the insect pests associated with stored groundnuts in Niger.

## 2 Materials and methods

An experiment was conducted at the Maradi Station of the National Institute of Agricultural Research – Niger (INRAN). Unshelled and shelled groundnuts were stored in 50 kg capacity PICS triple-layer bags (manufactured by Lela Agro Industries Ltd.; Kano, Nigeria) and in regular woven bags as the control. PICS bags consist of two high-density polyethylene (HDPE) liners of 80 μm wall thickness, fitted inside a woven polypropylene bag to enable handling. Naturally-infested shelled and unshelled groundnuts produced the previous season were purchased in a local market in Maradi. The experiment had two treatments: (1) PICS bags in four replicates and (2) woven bags in four replicates. Bags were filled with either 20 kg of unshelled groundnuts or with 10 kg of shelled groundnuts. Before filling the bags, each type of groundnut was physically and thoroughly mixed to ensure a uniform insect infestation. Replicate treatments were held on pallets in a storehouse under ambient local conditions. Although this does not allow control of temperature and humidity, it closely duplicates conditions under which groundnuts are stored in the real world of representative African farmers. Descriptions of how the PICS technology is used are available in several languages on the PICS website (https://picsnetwork.org/resources/?tab_id=Posters).

The experiments were initiated on 8th April 2015 and terminated on 27th October 2015 after a period of 202 days or 6.7 months. The following data was collected at the beginning and the end of the experiments: (i) Numbers of insects and damage levels were determined by randomly selecting three separate samples of 250 g of groundnuts from each bag (shelled and unshelled). Each sample was sieved and shelled (for unshelled groundnuts) to separate and count live adults and larvae. Pupae were counted as adults. One hundred seeds were picked randomly from each sample, weighed and then the number of seeds bearing eggs and holes determined by counting. (ii) To determine weight loss, a random sample of 100 grains or 100 pods were weighed at the beginning and the end of the experiment. The percent weight loss was estimated using the following formula-percentage weight loss = [(Initial weight -weight after 6.7 months)/(Initial weight)]*100. (iii) Germination was assessed as follows: (a) one hundred seeds from each sample were divided into four Petri dishes of 25 seeds each; the dishes were first lined with glass wool disinfected with 5% bleach; (b) distilled water was added to each dish to wet the wool; (c) germination was noted at up to 7 days. (iv) The oxygen (O_2_) and carbon dioxide (CO_2_) concentrations in each PICS bag were determined using a Mocon PAC Check^®^ Model 325 Headspace analyzer (Mocon, Minneapolis, MN). Measurements were made on each PICS bag before closing and at the end of the experiment using a hypodermic needle to penetrate into the interior of the bag though a small area from which the woven outer bag had been removed. (v) At the end of the experiments the two inner HDPE liners were examined to determine the number of holes made by insects and inspected for any other physical changes. (vi) In addition, we estimated the shelling percentage of the unshelled groundnuts by weight and by volume. Twelve samples of unshelled groundnuts of 200 g each and 12 samples of 250 ml each were used to estimate the shelling percentage for weigh and volume, respectively. The shelling percentage for weight was determined by dividing the weight after and before shelling x 100; while the shelling percentage for volume was calculated by dividing the volume after and before shelling x 100. The shelling percentage helped determine the efficiency of storing shelled compared with unshelled groundnuts in PICS bags.

Analysis of variance followed by the Student–Newman–Keuls (SNK) test was used to compare the means of infestation levels, insect damage and germination rate for groundnuts stored in PICS bags and woven bags (control) after 6.7 months of storage. The T test was used to compare the means O_2_ and CO_2_ levels in PICS bag at the beginning and the end of experiment. Statistical analysis was done with Statistical Package for the Social Sciences (SPSS) Version 16.0, IBM (Chicago, Illinois).

## 3 Results

Unshelled groundnuts: At the outset of the trial unshelled groundnuts had an average initial infestation of 7 living insects per sample of 250 g (Table 1). These were mainly adults of *T. castaneum* (50.74%), *C. cephalonica* larvae (40.29%) and *C. ferrugineus* adults (8.95%). After 6.7 months of storage, there was 100% mortality for each insect pest in PICS bags, while in the control woven bags the population of *T. castaneum* had increased 2.7-fold, *C. cephalonica* 6-fold, and *C. ferrugineus* 3.2-fold. There was also a shift in the relative numbers of the three species after 6.7 months of storage. *T. castaneum* now made up 33.6% of the insect population, *C. cephalonica* 59.5% and *C. ferrugineus* 6.9%.

**Table 1:** Insect infestation and damage to shelled and unshelled groundnut kept in 50 kg Purdue Improved Crop Storage (PICS) bags and woven bags filled with naturally infested grain (by *Tribolium castaneum, Corcyra cephalonica* and *Cryptolestes ferrugineus*) and stored for 6.7 months. Means followed by the same letter are not significantly different (LSD, 5%).

Groundnut types	Treatments	n	Living adults and larvae of insects in 250 g			100 grain/pods weight
			*T*. *castaneum*	*C. cephalonica*	*C. ferrugineus*	
Unshelled	Initial	24	3.4 ± 0.4 a	2.7 ± 0.3 a	0.6 ± 0.2 a	67.3 ± 0.4 a
	PICS bag after 6.7 months	12	0.0 ± 0.0 b	0.0 ± 0.0 b	0.0 ± 0.0 a	67.2 ± 0.5 a
	Woven bag after 6.7 months	12	9.2 ± 0.9 c	16.3 ± 1.8 c	1.9 ± 0.4 b	61.8 ± 0.4 b
	ANOVA		F = 51.635; *df* = 2/45; P < 0.01	F = 82.566; *df* = 2/45; P < 0.01	F = 10.835; *df* = 2/45; P < 0.01	F = 36.365; *df* = 2/141; P < 0.01
Shelled	Initial	24	0.2 ± 0.1 a	0.1 ± 0.0 a	0.0 ± 0.0 a	28.6 ± 0.0 a
	PICS bag after 6.7 months	12	0.4 ± 0.2a	0.0 ± 0.0 a	0.1 ± 0.1 a	28.6 ± 0.1 a
	Woven bag after 6.7 months	12	34.7 ± 4.0 b	0.2 ± 0.1 a	10.7 ± 2.9 b	20.4 ± 0.2 b
	ANOVA		F = 113.098; *df* = 2/45; P < 0.01	F = 1.071; *df* = 2/45; P = 0.351	F = 20.656; *df* = 2/45; P < 0.01	F = 1165.000; *df* = 2/141; P < 0.01

Shelled groundnuts: Shelled groundnuts had a mean initial infestation of 0.3 living insects per 250 g (Table 1). Of these, 66.7% were *T. castaneum* adults and 33.3% were larval *C. cephalonica*. At the end of the experiment, in the PICS bags, the densities of the pest were not different from those initially present. However, in woven bags the density of adults of *T. castaneum* was 174 times higher than the initial number while the density of *C. cephalonica* did not differ from that originally recorded. The proportions of the total insect pest population in the woven bags at the end of the experiment were 76.1% for *T. castaneum,* 0.44% for *C. cephalonica* and 23.5% for *C. ferrugineus*. Though not visibly evident at the outset of the experiment, after 6.7 months of storage *C. ferrugineus* averaged 11 adults per 250 g of shelled groundnut.

The weight of samples of shelled and unshelled groundnuts stored in PICS bags after 6.7 months of storage did not differ from that recorded at the beginning of the experiments (Table 1). By contrast, in woven bags we noted an average weight loss of 8.2% for unshelled groundnut and 28.7% for shelled groundnuts (Table 1). Unshelled groundnuts used in this experiment had an initial shelling percentage of 67.3± 1.6% in relation to the weight and 29.3± 4.6% in relation to the volume (data not presented). Germination rates at the end of the experiments for both unshelled and shelled groundnuts stored in PICS bags were comparable to those observed at the beginning of the trial (Table 2). By contrast, both types of groundnuts stored in regular woven bags had reductions in germination amounting to 14.9% for unshelled groundnuts and 64.8% for shelled groundnuts (Table 2).

**Table 2:** Germination rate of unshelled and shelled groundnut naturally infested, filled 50 kg Purdue Improved Crop Storage (PICS) bags and woven polypropylene bags and stored for 6.7 months. Means followed by the same letter are not significantly different (LSD. 5%).

Groundnut types	Treatments	n	Germination rate (%)
Unshelled	Initial	48	83.7 ± 1.6 a
	PICS bag after 6.7 months	24	81.1 ± 1.6 a
	Woven bag after 6.7 months	24	68.8 ± 2.1 b
Shelled	initial	48	87.8 ± 1.5 a
	PICS bag after 6.7 months	24	88.8 ± 2.6 a
	Woven bag after 6.7 months	24	23.0 ± 3.9 b

The initial mean oxygen levels of about 21% (v/v) decreased by 2% for unshelled and 5% for shelled groundnuts stored in PICS bags for 6.7 months (Table 3). Carbon dioxide was below the level of detection at the beginning of the experiment and increased only a little after 6.7 months of groundnut storage in PICS bags. At the end of the experiment, all PICS bags were inspected and no alterations or perforations were observed on any outer or inner HDPE liners (data not shown).

**Table 3:** Rate of oxygen and carbon dioxide in Purdue Improved Crop Storage (PICS) bags containing naturally infested shelled and unshelled groundnut stored for 6.7 months.

Groundnut types	Treatments	n	Level oxygen and carbon dioxide (v/v)	
			Oxygen	Carbone dioxide
Unshelled	Initial	4	20.6 ± 0,05	0.0 ± 0.0
	After 6.7 months	4	17.7 ± 0.3	0.4 ± 0.0
	Test t		(t = 22.81; *dl* = *6*; p < 0.01)	(t = 31.51; *dl* = *6*; p < 0.01)
Shelled	Initial	4	20.6 ± 0.1	0.0 ± 0.0
	After 6.7 months	4	14.9 ± 3.7	3.1 ± 1.9
	Test t		(t = 3.03; *dl* = *6*; p < 0.05)	(t = 3.25; *dl* = *6*; p < 0.05)

## 4 Discussion

Our results establish that PICS bags are effective at suppressing storage pests and maintaining grain quality during shelled and unshelled groundnut storage in the West African nation of Niger. *T. castaneum* and *C. cephalonica* species were the most important pests we found in naturally-infested groundnut stored for about 7 months. They are well-known pests of groundnut that affect food quality and seed viability (Nautiyal, 2002, Ranga Rao et al., 2010, Delobel and Matokot, 1990). After nearly 7 months of storage in PICS bags, pest population growth was suppressed in both shelled and unshelled groundnuts. When infested grain was stored for 6.7 months in woven bags, the population of *C. cephalonica* was suppressed in favor of *T. casteneum* in shelled groundnuts. *C. ferrugineus* was a minor pest at the beginning of the experiment in unshelled groundnuts and was not found in shelled groundnuts. *C. ferrugineus* is considered a secondary pest on groundnuts and is often found in association with other pests (Nautiyal, 2002, Sallam, 2008, Raoul and Léonard, 2013). It was observed at the end of the experiment that *C. ferrugineus* developed well when *T. castaneum* was thriving on shelled groundnut stored in woven bags.

Groundnuts (shelled and unshelled) stored in PICS bags experienced minimal weight loss, less than 1%. Shelled groundnuts stored in woven bags were damaged mainly by *T. castaneum* while the unshelled groundnuts was mainly damaged by *C. cephalonica.* While unshelled groundnuts had an initial higher insect infestation level (23 times) than shelled groundnuts, their weight loss was less affected at the end of the experiment. Weight loss of unshelled and shelled groundnuts kept in woven bags was 8.2% and 28.7%, respectively. Nautiyal (2002) reported that *T. castaneum* is a major pest of shelled groundnuts and can causes weight losses of 4.5%. In our experiments in Niger *T. castaneum* caused substantial damage to shelled groundnuts, more than 7 times that reported by Nautiyal (2002). The damage resulting from *C. cephalonica* is well documented for other crops. For example, with maize in India it leads to 7.46% weight loss after 2 months of storage (Meena et al., 2014). With cocoa beans in Ghana it causes weight loss of 13.34% after 4 months of storage (Azalekor, 1999). As is well known, the groundnut shell helps protect the seed from damage by storage insects. Some producer organizations recommend keeping groundnuts in the shell form when storage is for a period exceeding 6 months (http://www.rongead.org/IMG/pdf/BAI_stockage_arachide.pdf). *C. serratus*, despite its reputation for causing damage to stored groundnuts, was not observed in our experiment in Niger. However, several authors have reported its presence in the Sahelian zone (Aitken, 1975, Guèye et al., 2011). While storage in unshelled form helps farmers deal with insects, storing unshelled groundnuts uses substantially more volume in the container or stores. Storing groundnuts in shell increases the cost of storage by reducing the quantity of grain stored by a third. This can involve additional costs for buying bags and storage space. Therefore, the use of the PICS bag for shelled groundnut appears to be the most effective and economical way of storing groundnuts.

Oxygen levels in PICS bags decreased for both shelled and unshelled groundnuts after 6.7 months of storage, with the decrease more pronounced in shelled groundnuts. The levels of oxygen in this study were similar to those observed by Sudini et al. (2015) in an experiment conducted on groundnut pods in India but less than has been observed with other stored grains. The decrease in oxygen may explain the cessation of pest development. Since O_2_ and CO_2_ levels were not measured except at the start and end of the experiment, it is possible that O2 levels fell well below the final value observed and then, after the insect population had ceased activity or died, slow leakage of O2 back into the bag may have occurred. Further studies are needed to determine if this in fact occurred. It is well known that the conditions of hypoxia and hypercarbia disturb insect metabolism and can result in death (Hochachka, 1986, Weyel and Wegener, 1996, Ofuya and Reichmuth, 2002, Murdock et al., 2012). The higher level of oxygen in unshelled groundnuts may be explained by the bulkiness of the shells that create more air space compared to shelled groundnuts, which are more compact and contain less free airspace.

Germination of shelled and unshelled groundnuts kept in PICS bags for 6.7 months did not change – there was only a slight reduction of less than 3%. However, storage of groundnuts in woven bags caused germination reductions of up to 64.8% for shelled groundnuts. Nautiyal (2002) reported a reduction of 73% in germination when shelled groundnuts were infested by *T. castaneum*. These findings are consistent with the results of Sudini et al. (2015) who reported that PICS bags protected groundnut seed viability and seed weight under artificial infestation with *C. serratus*. Similar results have been noted on naturally-infested stores of Bambara groundnut and maize (Baoua et al., 2014a, Baoua et al., 2014b). The loss of germination recorded in the control treatments was almost certainly due to seed-embryos destruction by the insects. PICS bags performed better than Supergrain bags (another type of hermetic bag available to farmers in developing countries) for storing groundnuts (Harish et al., 2014). Supergrain bags enabled the development of *C. serratus* with a weight loss of 66.9% and 75.9% after 6 months of storage for shelled and unshelled groundnuts, respectively. We observed no perforations or other damage to the PICS HDPE liners. This suggests that the bags can be reused for two or more seasons, decreasing their overall cost and increasing the benefit to farmers.

In summary, naturally infested shelled and unshelled groundnuts can be safely stored in PICS bags, which arrest insect population growth and prevent associated grain degradation without affecting germination. Storing shelled groundnuts in PICS appear to be cost-effective.
